# Increased Stiffness in Aged Skeletal Muscle Impairs Muscle Progenitor Cell Proliferative Activity

**DOI:** 10.1371/journal.pone.0136217

**Published:** 2015-08-21

**Authors:** Grégory Lacraz, André-Jean Rouleau, Vanessa Couture, Thomas Söllrald, Geneviève Drouin, Noémie Veillette, Michel Grandbois, Guillaume Grenier

**Affiliations:** 1 Centre Hospitalier de l’Université de Sherbrooke Research Center (CRCHUS), Université de Sherbrooke, Sherbrooke, Quebec, Canada; 2 Department of Electrical and Computer Engineering, Faculty of Engineering, Université de Sherbrooke, Sherbrooke, Quebec, Canada; 3 Department of Pharmacology, Faculty of Medicine, Université de Sherbrooke, Sherbrooke, Quebec, Canada; 4 Department of Orthopedic Surgery, Faculty of Medicine, Université de Sherbrooke, Sherbrooke, Quebec, Canada; University of Louisville School of Medicine, UNITED STATES

## Abstract

**Background:**

Skeletal muscle aging is associated with a decreased regenerative potential due to the loss of function of endogenous stem cells or myogenic progenitor cells (MPCs). Aged skeletal muscle is characterized by the deposition of extracellular matrix (ECM), which in turn influences the biomechanical properties of myofibers by increasing their stiffness. Since the stiffness of the MPC microenvironment directly impacts MPC function, we hypothesized that the increase in muscle stiffness that occurs with aging impairs the behavior of MPCs, ultimately leading to a decrease in regenerative potential.

**Results:**

We showed that freshly isolated individual myofibers from aged mouse muscles contain fewer MPCs overall than myofibers from adult muscles, with fewer quiescent MPCs and more proliferative and differentiating MPCs. We observed alterations in cultured MPC behavior in aged animals, where the proliferation and differentiation of MPCs were lower and higher, respectively. These alterations were not linked to the intrinsic properties of aged myofibers, as shown by the similar values for the cumulative population-doubling values and fusion indexes. However, atomic force microscopy (AFM) indentation experiments revealed a nearly 4-fold increase in the stiffness of the MPC microenvironment. We further showed that the increase in stiffness is associated with alterations to muscle ECM, including the accumulation of collagen, which was correlated with higher hydroxyproline and advanced glycation end-product content. Lastly, we recapitulated the impaired MPC behavior observed in aging using a hydrogel substrate that mimics the stiffness of myofibers.

**Conclusions:**

These findings provide novel evidence that the low regenerative potential of aged skeletal muscle is independent of intrinsic MPC properties but is related to the increase in the stiffness of the MPC microenvironment.

## Introduction

Aging is inextricably associated with the loss of skeletal mass. Many factors contribute to this physiological process, including hormonal changes, malnutrition, chronic inflammation, and a decrease in the regenerative potential of muscle stem cells or myogenic progenitor cells (MPCs) [1]. Previous studies have shown that aged muscle contains fewer MPCs with impaired proliferative capacity and differentiation potential than adult muscle, and would explain the failure of aged muscle to regenerate successfully [2–4]. Changes in the microenvironment of the stem cell niche may also have a negative impact on MPCs [5–7]. This hypothesis is based on a number of seminal studies showing that alterations to Notch, insulin, FGF2, Wnt, and TGFβ, are involved in the fates of MPC during aging (reviewed in [8]). More recently, we showed that changes in the stiffness of the microenvironment of MPCs modulate the function required for myofiber repair [9].

The extracellular matrix (ECM), which affects the mechanical properties of the tissue microenvironment, directly influences the activity of various stem cells, including MPCs [10–13]. Changes in the composition of the ECM provide regulatory cues to MPCs and have an impact on their quiescence, activation, differentiation, and/or self-renewal [14]. Cells explore the mechanical properties of their environment by generating contractile forces through the actomyosin contractile machinery and then migrate or proliferate appropriately, a phenomenon called active mechanosensing [15].

In skeletal muscle, the ECM plays a major role in determining the stiffness of the entire microenvironment [16–18]. Alterations to muscle ECM components such as collagen and advanced glycation end-products (AGE) may be determinants of the stiffness-induced decline in force transmission that occurs with age [16, 19]. Given that ECM is a major component of the MPC niche, we propose that modifications to the ECM with aging may promote stiffness-associated MPC behavior that in turn may alter the regenerative potential of the muscle.

We showed that freshly isolated individual myofibers from aged muscle have fewer quiescent MPCs and are less proliferative than myofibers from adult muscle. The proliferative state of MPCs was impaired in damaged cultured myofibers from aged muscle. Using AFM-based indentation experiments, we also showed that the differences between the MPC activity of adult and aged muscle are due to an increase in the stiffness of the microenvironment caused by an increase in ECM content and crosslinking. To our knowledge, the present study is the first to use individual myofibers, a physiological model that preserves the anatomical satellite cell niche, to show that changes to the biomechanical microenvironment have an impact on MPC behavior during aging.

## Materials and Methods

### Animals

Four-month-old (adult) and twenty-four-month-old (aged) male C57Bl/6 mice (Charles River, Canada) were housed in our animal colony. The study was carried out in strict accordance with the recommendations of the Canadian Council on Animal Care guidelines. The protocol was approved by the Committee on the Ethics of Animal Experiments of the University of Sherbrooke (Permit Number: 133-14B). The mice were anesthetized (isoflurane; Abbott Laboratories, Canada) prior to being euthanized with CO_2_, and all efforts were made to minimize suffering. The quadriceps (Quad), extensor digitorum longus (EDL), and tibialis anterior (TA) muscles were then harvested.

### Myofiber and myoblast isolation and culture

Single myofibers from EDL muscles were isolated by collagenase digestion as previously described [20], with slight modifications. Briefly, EDL muscles were digested with collagenase for 1 h at 37°C with frequent gentle agitation. Myofibers were harvested individually at room temperature using horse serum (HS)-coated Pasteur pipettes. Following several rinses with proliferative medium (Ham’s F10 supplemented with 20% fetal bovine serum (FBS; Hyclone), 1% antibiotics (penicillin/streptomycin; Wisent), and 2.5 ng/ml of bFGF (Invitrogen), the myofibers were incubated with collagenase for 10 min, transferred to proliferative medium, and incubated at 37°C in a 5% CO_2_ humidified incubator for 6 days. Intact myofibers and myofibers that collapsed (damaged) when cultured were either fixed in 4% paraformaldehyde (PFA) for 10 min, rinsed in PBS, and stored at 4°C for immunofluorescence studies, or were triturated with HS-coated Pasteur pipettes to isolate peripheral MPCs (or myoblasts). Isolated myoblasts were then enriched and were amplified by four passages in proliferative medium in collagen-coated petri dishes.

To evaluate their proliferative and differentiation capacities, primary myoblasts were seeded at 2000 cells/cm^2^ and were counted after 72 h in culture using a Neubauer hemocytometer. Cumulative cell population doubling (CPD) was calculated as described previously [21].

For differentiation assays, the medium of confluent myoblasts was replaced with differentiation medium consisting of DMEM supplemented with 5% HS. After 3 days, the cells had fused to form multinucleated myotubes. Differentiation efficiency was evaluated by assessing the fusion index, which is expressed as the percentage of myosin heavy chain-positive cells that contain more than two nuclei.

### Histology and immunofluorescence

TA muscles from adult and aged mice were fixed in formalin and were embedded in paraffin. Four-micrometer sections were cut using a HM325 microtome (Micron, Germany). The sections were rehydrated and were stained with Masson trichrome, as described previously [20]. Images were acquired using a Nanozoomer 2.0 RS series slide scanner (Hamamatsu, Japan).

For immunofluorescence staining, freshly isolated and cultured myofibers as well as primary myoblasts were fixed in 4% PFA at 4°C for 10 min and were then permeabilized and blocked in PBS containing 10% goat serum, 1% BSA, and 0.2% Triton X-100. Cell preparations were incubated with mouse anti-Pax7 (1:3; DSHB, USA), mouse anti-myosin heavy chain (1:10; MF20, DSHB), or rabbit anti-MyoD (1:1500; C-20, Santa Cruz Biotech, USA) primary antibodies. After several rinses in PBS-Tween 20, the samples were incubated with Alexa Fluor 488-conjugated goat anti-mouse IgG (1:1000), Alexa Fluor 488-conjugated goat anti-rabbit IgG (1:1000), Alexa Fluor 594-conjugated goat anti-rabbit IgG (1:1000), or Alexa Fluor 594-conjugated goat anti-mouse IgG (1:1000) secondary antibody (Invitrogen, Canada). Samples in which the primary antibodies were omitted served as controls. Cell nuclei were labeled with DAPI (Sigma-Aldrich, Canada). An Axioskop 2 phase-contrast/epifluorescence microscope (Carl Zeiss, Inc., USA) was used to examine myofiber staining, and a TE2000e inverted microscope (Nikon Instruments Inc., USA) was used to examine the cell preparations. The photomicrographs were processed using Image Pro software (Media Cybernetics, USA).

### Measurement of muscle collagen content

Collagen content was determined by measuring hydroxyproline content using colorimetric assay kits (Biovision Inc., USA). Flash-frozen Quad muscles were crushed and dehydrated in an evaporator. Muscle samples (10 mg dry weight) were homogenized and were then hydrolyzed for 4 h at 110°C in 6 N HCl. The HCl was allowed to evaporate overnight. The preparation was then homogenized in 4 N NaOH, treated with chloramine T (Sigma-Aldrich) for 20 min at room temperature, and incubated in a solution of p-dimethylaminobenzaldehyde for 7 min at 65°C. Trans-4-hydroxy-L-proline solutions (Sigma-Aldrich) were used as a standard. Hydroxyproline concentrations were determined at 560 nm using a ThermoMax microplate reader (Molecular Devices, USA), and the results were normalized to dry weight [22]. Hydroxyproline content was converted to the percentage of collagen by multiplying the amount of hydroxyproline by a previously established factor (7.46) [23].

### Measurement of advanced glycation end products

Flash-frozen crushed Quad muscle samples (10 mg) were digested in a solution of 20 mg/mL proteinase K (Invitrogen) for 24 h at 55°C and were then heat inactivated. The concentration of advanced glycation end products (AGEs) was determined using OxiSelect Advanced Glycation End Product (AGE) Competitive ELISA kits (Cell Biolabs, USA) according to the manufacturer's protocol. AGE content was normalized to the total protein content determined using Bradford's method (Bio-Rad, Canada).

### Quantification of myofiber and muscle section stiffness by atomic force microscopy

Myofibers were isolated from the EDL muscles of WT mice using the procedure described above. Intact myofibers were either used directly for stiffness evaluations or were incubated in a proliferative medium for 6 days. Intact and damaged myofibers were transferred into 6-well plates with a piece of adhesive tape in the bottom of each well. The plates were centrifuged at 400 *g* for 10 min at room temperature to attach the myofibers to the tape. The pieces of tape with the attached myofibers were then transferred into petri dishes and were immersed in a CO_2_-independent medium (DMEM L15; Wisent) for stiffness quantifications. To evaluate differentiated myogenic cell stiffness, the C2C12 differentiation medium was replaced with DMEM L15. Stiffness was quantified with an AFM-based indentation approach using a custom-built force measurement device similar to the AFM setup described elsewhere [24]. Our setup was mounted on an Observer Z1 inverted microscope (Carl Zeiss, Germany) that allowed the cantilever tip to be precisely positioned above the myofiber. Prior to each experiment, the deflection sensitivity was determined in PBS in a plastic petri dish containing no myofibers or myotubes. The nominal spring constant of the silicon nitride cantilever (provided by the manufacturer) was used to convert the photodiode signal into a force value (k_nom_ = 0.05 N/m, MLCT; Bruker AFM Probes, USA). The tip of the cantilever was positioned in the middle of each myofiber or myotube, and three force-indentation curves were collected. The data were analyzed using an in-house code in Matlab (MathWorks, USA) based on the algorithm of Crick *et al*. [25]. A stepwise Young’s modulus was extracted from each force-indentation curve using a modified Hertz model for a four-sided pyramidal indenter [26]:

$$F\;=\;\frac{E}{1-\nu^{2}}\;\frac{\;\left(tan\;\alpha \right)}{\sqrt{2}}\delta^{2}$$

where F is the indentation force, E is Young’s modulus, ν is Poisson’s ratio (set to 0.5 for isotropic incompressible materials), δ is the indentation, and α is the face angle of the pyramid (17.5° for our cantilever). The mean of three Young’s modulus values was calculated for each myofiber or myotube and was used as an expression of stiffness.

TA and Quad muscles were embedded in 2% low melting point agarose, and 100-μm-thick longitudinal muscle sections were obtained using a Vibratome 3000 Plus (Vibratome, USA). Tissue stiffness was measured as described above for myofibers.

### Synthesis of stiffness-tunable polyacrylamide gels

Stiffness-tunable gel substrates with different stiffness values (0.5, 2.0, and 18 kPa) were synthesized using published methods [27, 28]. Briefly, 25-mm^2^ round glass coverslips were immersed in 0.1 M NaOH and were placed on a 80°C hot plate until the NaOH evaporated. The coverslips were coated with 3-aminopropyltriethoxysilane (APES), which allows gel adhesion, in a nitrogen-filled tent. The coverslips were rinsed several times with distilled water and then with 0.5% glutaraldehyde in PBS. Glass slides were treated in parallel with 10% dichlorooctamethyltetrasiloxane (SurfaSil) in chloroform for 10 min. Excess SurfaSil was wiped off, and the glass slides were rinsed with distilled water. Gel substrates were synthesized by mixing 40% acrylamide and 2% bis-acrylamide stock solutions with distilled deionized water to obtain the required stiffness. The mixtures were degassed under vacuum for 15 min. The substrates were polymerized by adding 5 mg/ml of ammonium persulfate and Temed (1:1000) N-hydroxysuccinimide. Twenty-five μl of gel solution was quickly sandwiched between a pre-treated glass slide and a pre-treated coverslip. The polymerized gels were rinsed with PBS and were collagen-coated overnight to allow cell adhesion. MPCs (1750 cells/cm^2^) were plated on the sandwiches and were cultured in proliferative medium.

### Statistics

All data are expressed as means ± standard error of the mean (SEM). An unpaired student *t-*test or a one-way ANOVA with a Friedman post-test was used to assess statistical significance between groups, as indicated. *P*<0.05 was considered to be statistically significant. Statistical values were obtained using GraphPad Prism 6.0 software (GraphPad Software Inc., USA).

## Results

### Aged myofibers exhibit altered MPC activity

**Fig 1:**
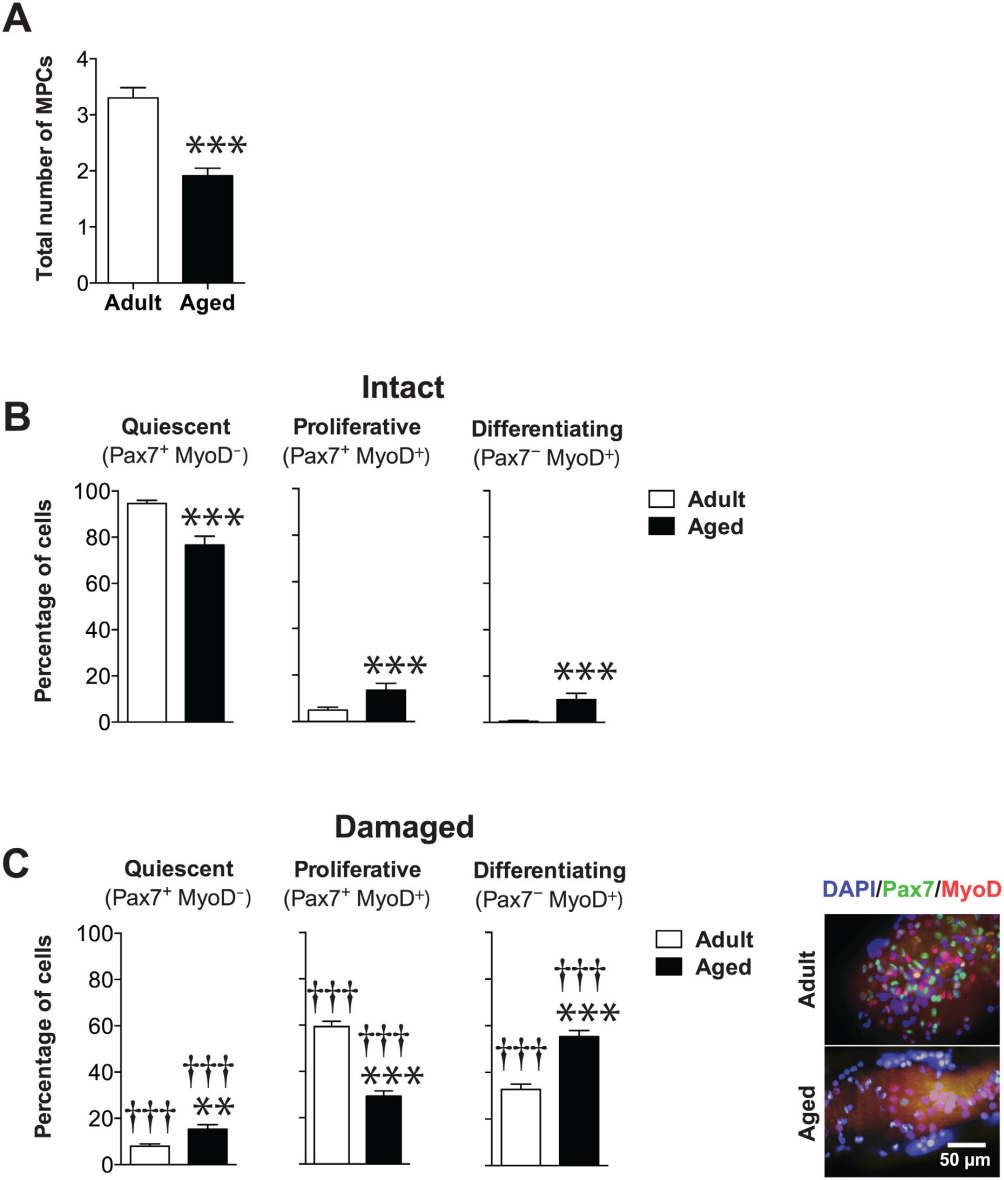
MPCs in aged myofibers display decreased myogenic activity. **(A**) Histograms showing the total number of MPCs per myofiber on 5–109 freshly isolated and cultured (6 days) myofibers from adult and aged mice (*n* = 5 mice per group). **(B, C)** Histograms showing the percentage of quiescent SCs (Pax7^+^MyoD^−^) and proliferating (Pax7^+^MyoD^+^) and differentiating MPCs (Pax7^−^MyoD^+^) per myofiber on intact **(B)** and damaged **(C)** myofibers. The Pax7 and MyoD proteins were immunostained as shown in the photomicrograph on the right. The count includes all parts and depths of the myofibers. The representative image on the right shows that Pax7 and MyoD proteins are expressed at lower levels in aged myofibers. **p<0.01 and ***p<0.0001 versus adult. ^†††^ *p*<0.001 versus intact counterparts. All data are expressed as means ± SEM.

We used freshly isolated intact myofibers immunostained with Pax7 and MyoD antibodies to obtain an accurate assessment of the MPC activity in aged skeletal muscle. We first examined the total number of MPCs on freshly isolated myofibers from 4- and 24-month-old male C57Bl/6 mice. Aged myofibers contained approximately 30% fewer MPCs (*p*<0.0001) than adult myofibers (Fig 1A).

We further investigated the phenotypes of the MPCs by determining the percentage of quiescent (Pax7^+^MyoD^−^), proliferative (Pax7^+^MyoD^+^), and differentiating (Pax7^−^MyoD^+^) cells, as described previously [29–32]. We found that 95±1% of the MPCs in adult myofibers were in a quiescent state compared to 77±4% in aged myofibers. The remaining cells were in the proliferative (14±3% vs. 5±1%) or differentiated state (0±0% vs. 10±3%) (Fig 1B).

We further investigated whether there was any difference in the MPC phenotype from adult vs. aged myofibers. After 6 days of culture, the percentage of quiescent cells dropped significantly for both adult and aged damaged myofibers compared to freshly isolated myofibers, with only 8±1% and 15±2%, respectively, remaining quiescent (Fig 1C). The percentage of proliferative cells was significantly higher in adult damaged myofibers (59±2%) than in aged ones (29±2%). The percentage of differentiating cells was also significantly lower in adult (33±2%) than in aged muscles (55±3%), suggesting that MPCs in aged damaged myofibers are more likely to differentiate.

### Cultured MPCs from adult and aged myofibers have similar proliferative and differentiation levels

It is well known that MPC phenotypes are strongly dependent on their microenvironment [3, 9, 33]. On the other hand, MPCs are quick to respond to their environment. We thus determined whether the differences in the phenotype resulted from a change in the microenvironment or from the intrinsic properties of the cells. We focused on the activation and proliferation processes, since the effect of stiffness on the differentiation into myotubes has already been characterized [34]. We first performed a cumulative population doubling experiment with primary myoblast cell lines derived from adult and aged myofibers. We did not observe any significant alteration in the proliferation potential of myoblasts due to aging (S1A Fig). We also characterized the differentiation activity of the MPCs by determining the fusion index. The differentiation potentials of MF20^+^ adult and aged cells were similar, as shown by the percentages of cells harboring more than two nuclei (54±2% vs. 53±1%) (S1B Fig). These results strongly suggested that an alteration in MPC behavior on myofibers is likely due to their microenvironment.

### Aging is associated with increased skeletal muscle stiffness

**Fig 2:**
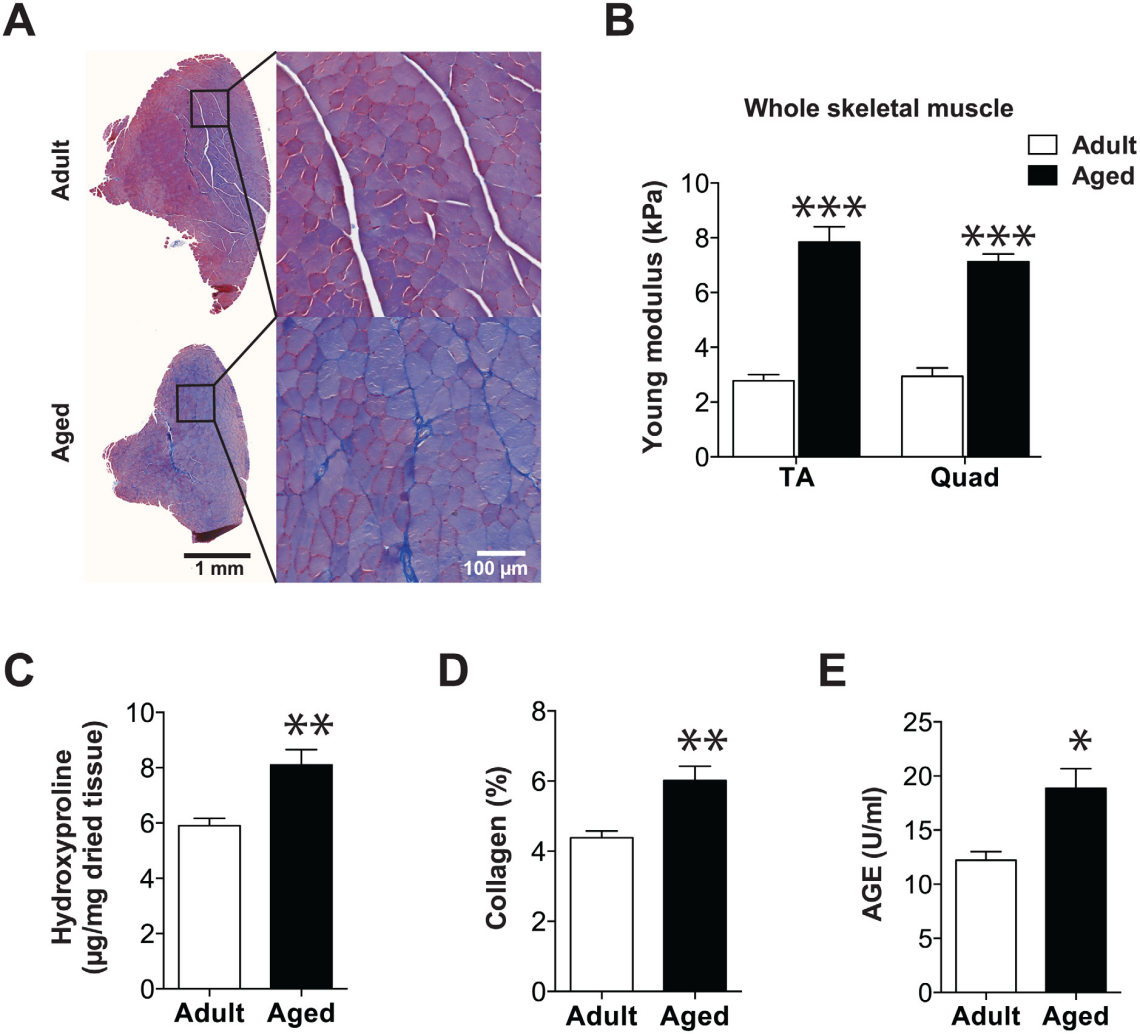
Aging is associated with increased skeletal muscle stiffness. **(A)** Representative images of Masson trichrome-stained TA sections from adult and aged male C57Bl/6 mice. **(B)** Graph showing the quantification of whole muscle stiffness from 100-μm-thick slices of TA and Quad muscles from adult (*n* = 4) and aged (*n* = 7) mice. Ten measurements per slice with three force-indentation curves collected per measurement were performed on 3–5 sections for each mouse. (**C**) Graph showing the biochemical quantification of total hydroxyproline content of Quad muscles from adult (*n* = 5) and aged (*n* = 5) mice. **(D)** Graph showing hydroxyproline values converted to collagen content. (**E**) Measurement of total advanced glycation end-product (AGE) content of Quad muscles from adult (*n* = 5) and aged (*n* = 5) mice. *p<0.05; **p<0.01; ***p<0.0001 versus adult group. All data are expressed as means ± SEM.

The MPC microenvironment is strongly associated with the basal lamina, which is made up of ECM. Since ECM deposition determines stiffness, which drives the fate of stem cells [10, 14, 16], we verified whether ECM deposition is altered in aged muscle. TA muscle sections stained with Masson trichrome showed that more collagen is deposited between myofibers in aged muscle than in adult muscle (Fig 2A). We then quantified Young’s modulus by AFM on longitudinal sections of adult and aged TA and Quad muscles. Excessive ECM deposition in aged muscle was correlated with a significant 3-fold increase in the stiffness of both TA and Quad muscles (Fig 2B). This was accompanied by a significantly higher hydroxyproline content (~30%), a marker of collagen-modified protein (Fig 2C). Collagen can be reticulated (cross-linked) by AGE, which also alters the stiffness of the ECM [35] (Fig 2D). AGE levels were also higher (65%) in aged than in adult muscles (Fig 2E). Altogether, our results showed that aging is associated with an accumulation of cross-linked collagen in skeletal muscle, which is responsible for the increase in stiffness.

### Damaged aged myofibers display robust stiffness

**Fig 3:**
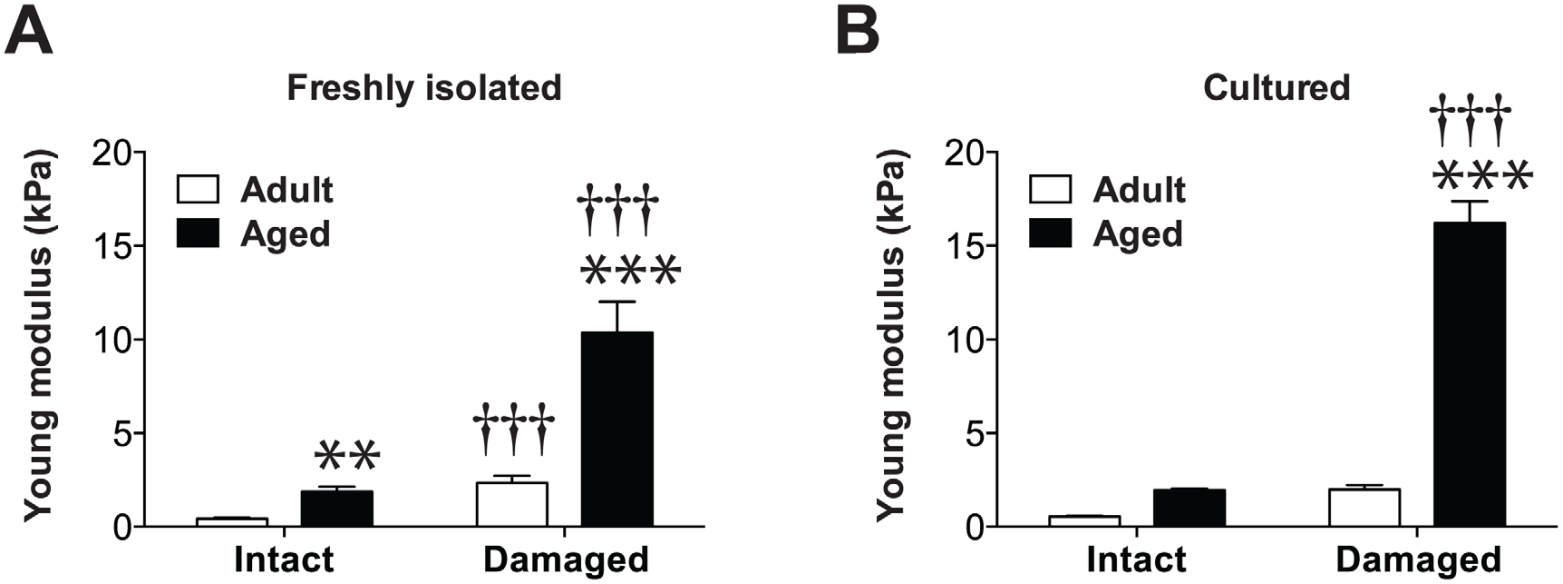
Damaged myofibers from aged mice display robust stiffness that impairs MPC activity. Graphs showing Young’s modulus values (kPa) and stiffness measurements of freshly isolated (**A**) and cultured intact and damaged myofibers (**B**) from adult (*n* = 3) and aged (*n* = 5) muscles. One measurement (three force-indentation curves collected for each measurement) per myofiber was performed on 6–50 myofibers. **p<0.01; ***p<0.0001 versus adult counterpart. ^†††^ *p*<0.001 versus intact counterpart. All data are expressed as means ± SEM.

The stiffness of the microenvironment directly modulates the myogenic activity of MPCs [10, 34, 36]. Given that MPCs are located under the basal lamina surrounding myofibers, we measured the stiffness of individual myofibers from adult and aged mice by AFM. Our results indicated that freshly isolated intact aged myofibers from aged mice are significantly stiffer than freshly isolated intact myofibers from adult mice (1.9±0.3 vs. 0.4±0.1 kPa) (Fig 3A). We then explored the stiffness of myofibers that had been cultured for 6 days. Our results revealed that the stiffness of cultured intact adult and aged myofibers is not statistically different from those of freshly isolated adult and aged myofibers. The greatest differences were observed between freshly isolated (2.3±0.4 vs. 10.4±1.6 kPa, respectively) and damaged (2.0±0.2 vs. 16.2±1.2 kPa, respectively) adult and aged myofibers (Fig 3B).

### Age-associated myofiber stiffness in vitro recapitulates in vivo MPC behavior

**Fig 4:**
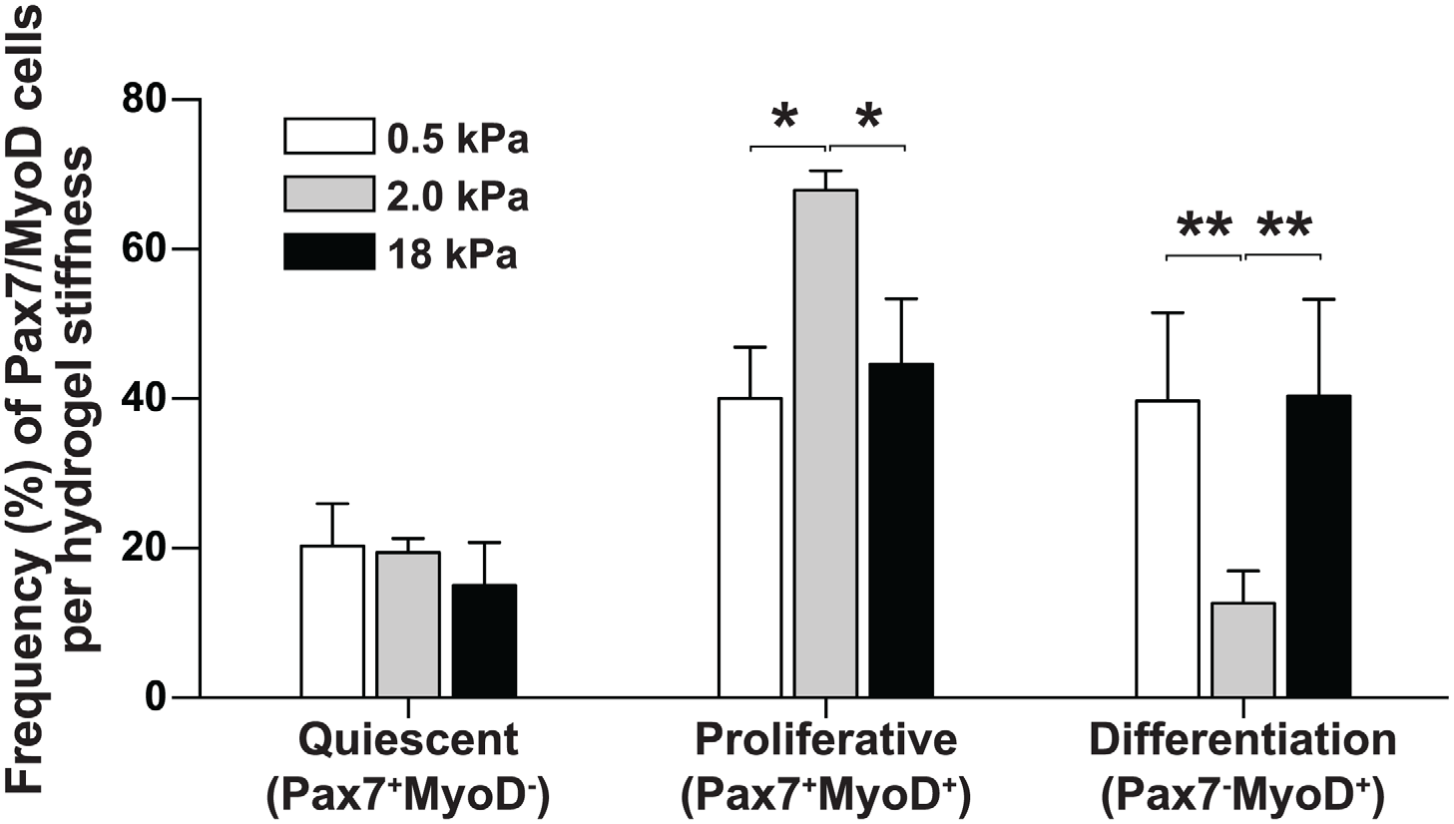
Hydrogels mimicking myofiber stiffness recapitulate MPC behavior. Histograms showing the frequency of quiescent, proliferative, and differentiating MPCs (Pax7^+^MyoD^-^, Pax7^+^MyoD^+^ and Pax7^−^MyoD^+^, respectively), as a function of hydrogel stiffness (0.5, 2.0, and 18 kPa). Primary myoblasts from adult (*n* = 5) mice were used for this experiment. Cells were plated and grown for 48 h. The cells were then fixed and stained with DAPI and immunostained with Pax7 and MyoD antibodies. We counted the number of cells in five random fields in four plates for each stiffness preparation. All data are expressed as means ± SEM (*p<0.05; **p<0.001).

To assess the contribution of stiffness to an alteration in MPC behavior, we cultured and characterized MPCs on substrates mimicking the stiffness of adult (0.5 kPa) and aged (2 kPa) fresh intact myofibers, and to adult (2 kPa) and aged (18 kPa) cultured damaged myofibers (Fig 4). As expected, the frequency of proliferative MPCs (Pax7^+^MyoD^+^) was 1.7-fold higher (*p* = 0.0155) with the 2 kPa substrate than with the 0.5 kPa substrate, which was consistent with the increased proliferation observed in freshly isolated intact aged versus adult myofibers. On the other hand, we observed a 1.5-fold decrease (*p* = 0.0159) in the percentage of proliferating MPCs grown on the 18 kPa hydrogel, which was consistent with the low percentage of proliferative MPCs observed in aged damaged myofibers. In this case, a higher percentage of MPCs have adopted a differentiating phenotype at 18 kPa, suggesting that MPCs are less proliferative and more likely to differentiate in aged versus damaged myofibers (3.3-fold increase; *p* = 0.0079). Lastly, we observed no significant difference in the proportion of Pax7^+^MyoD^-^ cells (quiescent) grown on the 0.5, 2.0, or 18 kDa hydrogels, which might be due to the intrinsic activation of MPCs grown in culture.

## Discussion

Aging is associated with a decrease in the regenerative capacity of skeletal muscle [1]. A better understanding of the mechanisms modulating the fate and function of MPCs is vital for the development of new tools to prevent the skeletal muscle loss that occurs with aging. In the present study, we characterized the impact of biomechanical changes in the aged muscle microenvironment on MPC behavior. We used myofibers and myoblasts derived from primary cultures as physiological models and showed that the increase in stiffness of aged damaged myofibers reduces the proliferative capacities of MPCs, which can impair the regenerative capacity of aged muscle.

We showed that freshly isolated aged myofibers contain fewer MPCs, especially quiescent satellite cells (Pax7^+^MyoD^−^), than adult myofibers. These results were comparable to those of other studies showing that the relative number of satellite cells decreases with age, pointing to a lower rate of self-renewal or to asymmetrical division [3, 4]. We recapitulated the MPC behavior observed on myofibers from adult and aged muscles using stiffness-tunable hydrogels and observed that there was a higher proportion of differentiating MPCs in aged damaged myofibers (18 kPa) than in adult damaged myofibers (2 kPa). This is consistent with the results obtained with MPCs grown on the 2 and 18 kPa hydrogels and indicated that there is a more committed MPC phenotype in aged myofibers. It is possible that an increase in the stiffness to 2 kPa of adult damaged myofibers is beneficial for the activation/proliferation of MPCs. On the other hand, an increase in the stiffness to 18 kPa, as observed in damaged aged myofibers, would be deleterious for the proliferation of MPCs but would favor differentiation. This may be one explanation for the decline in the regenerative capacity of aged skeletal muscle.

A growing body of evidence suggests that the stem cell niche serves as an environment in which stem cells respond to extrinsic stimuli associated with muscle growth and repair [37–42] and that the mechanisms involved are negatively regulated by aging [7]. As we showed, when MPCs are dissociated from their niche, the proliferation and differentiation potentials of MPCs from aged mice are similar to those of MPCs from adult mice, which lends support to the importance of the MPC niche.

Our group has recently shown that the stiffness of the MPC microenvironment may have a significant impact on their potential for myofiber repair through an alteration to their proliferation activity and maintenance [9]. The composition of the ECM affects the mechanical properties of the tissue microenvironment, which in turn influences the activity of stem cells [10, 11]. Given that the ECM plays a major role in the increase in stiffness that occurs with age [16, 43], some authors have suggested that there is a correlation between the increase in collagen deposition and the increase in muscle stiffness [19], with AGE playing a major role in glycation and collagen reticulation [16, 35, 44]. Changes in the composition of the ECM during aging would thus provide regulatory cues to stem cells, modulating their quiescence, activation, differentiation, and/or self-renewal [14]. In the present study, we confirmed that the increase in collagen deposition in the muscles of aged mice is correlated with an increase in hydroxyproline and AGE levels. These results reinforce the notion that the ECM undergoes qualitative and quantitative modifications with aging that would alter the myofiber repair process. Notably, excessive collagen deposition in muscle leads to fibrosis, a major feature of aging and dystrophic/reparative disorders [44–47]. This also corroborates our previous findings showing that there is a decrease in the expression of genes coding for components of the ECM that are required for the maintenance of the stem cell niche in aged muscle [29].

## Conclusion

Our results provided novel evidence that the low regenerative capacity of aged skeletal muscle is independent of intrinsic MPC properties, but is rather linked to the myofiber microenvironment. In addition to other biochemical factors reported previously, we found that an alteration to the mechanical stiffness of the ECM in aged skeletal muscle impair MPC activities. The biomechanical environment of MPCs and the pathways that contribute to muscle repair should thus be further investigated.

## Supporting Information

S1 Fig Age does not alter the intrinsic proliferative and differentiating potentials of myofiber MPCs. **(A)** Graph showing the cumulative population doubling of primary MPCs (or myoblasts) isolated from adult (*n* = 3) and aged (*n* = 3) mice. The proliferation measurements were performed on three passages of each cell line. **(B)** Graph showing the fusion index of adult (*n* = 3) and aged (*n* = 3) MPC cell lines. The fusion index was determined by counting myosin heavy chain-positive cells that contained more than two nuclei. Representative images of myosin heavy chain (MF20, red) immunostaining, with nuclei in blue (DAPI), in cells used for the fusion index calculations. All data are expressed as means ± SEM.
(EPS)
