# Correction: Increased Stiffness in Aged Skeletal Muscle Impairs Muscle Progenitor Cell Proliferative Activity

**DOI:** 10.1371/journal.pone.0167661

**Published:** 2016-12-01

**Authors:** Grégory Lacraz, André-Jean Rouleau, Vanessa Couture, Thomas Söllradl, Geneviève Drouin, Noémie Veillette, Michel Grandbois, Guillaume Grenier

The fourth author's name is spelled incorrectly. The correct name is: Thomas Söllradl.

The correct citation is: Lacraz G, Rouleau A-J, Couture V, Söllradl T, Drouin G, Veillette N, et al. (2015) Increased Stiffness in Aged Skeletal Muscle Impairs Muscle Progenitor Cell Proliferative Activity. PLoS ONE 10(8): e0136217. doi:10.1371/journal.pone.0136217.
